# Overcrowding in a neonatal intermediate care unit: impact on the incidence of multidrug-resistant gram-negative organisms

**DOI:** 10.1186/s12879-019-3981-8

**Published:** 2019-04-29

**Authors:** Doris Fischer, Rolf L. Schlößer, Volkhard A. J. Kempf, Thomas A. Wichelhaus, Thomas Klingebiel, Sabine Philippi, Linda Falgenhauer, Can Imirzalioglu, Udo Dahl, Christian Brandt, Claudia Reinheimer

**Affiliations:** 1 Department of Pediatrics, Division of Neonatology, University Hospital Frankfurt, St. Vincenz Hospital, Auf dem Schafsberg, 65549 Limburg, Germany; 2 Institute of Medical Microbiology and Infection Control, University Hospital Frankfurt, 60590 Frankfurt at the Main, Germany; 3 University Center for Infectious Diseases (UCI), University Hospital Frankfurt, 60590 Frankfurt at the Main, Germany; 4 Justus Liebig University, Institute of Medical Microbiology, and German Center for Infection Research (DZIF), Partner site Giessen-Marburg-Langen, Giessen, Germany

**Keywords:** Multidrug-resistant gram-negative bacteria, Overcrowding, Neonatal intermediate care unit, Nurse to patient ratio, Infection control, Microbiological surveillance

## Abstract

**Background:**

Overcrowding, reduced nurse to patient ratio, limited distance between incubators and absence of microbiological surveillance have been shown to promote spread of multidrug-resistant gram-negative organisms (MDRGN) in patients with birthweight < 1500 g. Patients > 1500 g treated on an intermediate care unit are unrepresented in recent literature. We therefore intended to present data obtained from a short-term overcrowded neonatal intermediate care unit (NIMCU) at a level III (international categorization) perinatal center at University Hospital Frankfurt, Germany.

**Methods:**

During a 25 day overcrowding (OV) and 28 day post-overcrowding period (POST-OV) on NIMCU, epidemiological data obtained from continuously hold microbiological surveillance were investigated and compared to the last 12 months of ward-regular bed occupancy preceding OV (PRAE-OV).

**Results:**

During OV, the number of patients simultaneously treated at the NIMCU increased from 18 to 22, resulting in a reduced bed-to-bed space. Nurse: patient ratio was 4:22 during OV compared to 3:18 during PRAE-OV. Cumulative incidence of MDRGN was 4.7% in OV and 2.4% POST-OV compared to 4.8% to PRAE-OV, respectively, without any significant variations. During OV and POST-OV, septic episodes due to MDRGN were not observed. In one case, potential nosocomial transmission of *Enterobacter cloacae* resistant to Piperacillin and 3rd/4th generation cephalosporins was observed.

**Conclusions:**

Prevention of nosocomial spread of MDRGN in an overcrowded NIMCU is based on staff’s diligent training and adequate staffing. Concise microbiological surveillance should be guaranteed to escort through overcrowding periods. In our setting, impact of bed-to-bed distance on MDRGN transmission seemed to be less strong.

## Background

The rapid global spread of multidrug-resistant gram-negative organisms (MDRGN) is a serious global health risk, associated to several factors, e.g. traveling to high-prevalence countries (HPC) for MDRGN or contact to healthcare-systems in HPC [1–8]. Since MDRGN frequently cause community and healthcare-associated infections in almost all countries and medical disciplines [9–11], efforts to prevent infections caused by MDRGN should therefore not only address adequate usage of antibiotics and the development of strategies to prevent spread of MDRGN, but also on enhancement of the knowledge of MDRGN transmission, especially in hospital settings. Overcrowding has been shown to promote the spread of the Middle East Respiratory Syndrome coronavirus (MERS-CoV) in medical wards [12–14]. Overcrowding was mentioned as well as one major factor in the spread of severe acute respiratory syndrome (SARS) due to SARS-coronavirus (CoV) in healthcare workers [15]. Transmission of methicillin-resistant *Staphylococcus aureus* (MRSA) is also promoted by overcrowding [16, 17]. In neonatal units overcrowding has also been shown to foster the spread of gram-negative bacteria and central venous lines (CVC) associated bloodstream infections [18]. Overcrowding therefore should be considered to promote transmission and spread of MDRGN on neonatal units.

Although overcrowding in medical wards should be impeded in order to prevent transmission of multidrug-resistant pathogens, it however might be inevitable sometimes [19, 20]. Due to inalienable reconstruction in June / July 2016, number of simultaneously treated patients at the neonatal intermediate care unit (NIMCU) at the University Hospital Frankfurt (UHF) increased under identical spatial conditions, resulting in an overcrowed NIMCU. This study highlights epidemiological, microbiological and neonatological management in order to give insight of infection control in a short-term overcrowded NIMCU.

## Methods

### Definitions

Increase of hospital mortality has been attributed to overcrowding [18–21], however, no detailed definition has been formulated. We followed the definition by *Gordon* et al [22].

PRAE-OV was defined as the one-year-period prior to the overcrowding period with NIMCU running under regular bed-occupancy. PRAE-OV was introduced to calculate the average incidence of MDRGN on NIMCU which is the epidemiological benchmark for OV and the post-overcrowding observational period (POST-OV), the latter being defined as the 28-day-period following OV.

Nosocomial transmission was given, if neonatal as well as the corresponding mother’s MDRGN screening was negative on day of admission and the following neonatal MDRGN screening turned positive. Vertical transmission was given, if baby and mother were tested positive for identical MDRGN species and phenotypical resistance pattern at any location.

MDRGN have previously been defined as *Enterobacteriaceae* with extended spectrum beta–lactamase (ESBL)–phenotype, and *Enterobacteriaceae, Acinetobacter baumannii* and *Pseudomonas aeruginosa* resistant against piperacillin, any 3rd/4th generation cephalosporin, and fluoroquinolones +/− carbapenems [7, 25].

### Conditions of overcrowding and the university hospital Frankfurt

The pediatric intensive care unit (PICU) at UHF had been under construction from calendar week (CW) 23 to CW 26 in 2016. Given the nearly continuous maximally occupancy of NICU beds at any paediatric hospital in the Rhine-Main area, transferring neonates to other NICU/NIMCUs was less likely. Therefore, all neonates meeting the entry criteria for NIMCU were transferred from PICU to NIMCU at UHF. This period took 25 days under which the ward was scheduled under overcrowded conditions. During OV, number of beds increased from *n* = 18 to *n* = 22; bed to bed-distance decreased to 1.0–1.2 m. Considering that distance between NICU and NIMCU beds has been recommended to be 2 m at least [23–26], OV resulted in a infectiologically risky setting.

### Detection of multidrug-resistant gram-negative organisms and molecular resistance analysis

All laboratory testing procedures were performed under strict quality–controlled criteria (ISO 15189:2007 standards) and as previsouly described [7]. Detection of genes encoding carbapenemases are routinely performed via PCR analysis and subsequent sequencing from carbapenem resistant *Enterobacteriaceae* including the genes for carbapenemases genes NDM, VIM, IMP, OXA–48, and KPC [27]. Identification of *Acinetobacter baumannii* within the *Acinetobacter calcoaceticus* – *Acinetobacter baumannii* (ACB) complex was done by a molecular *in house* procedure for detection of the species-specific carbapenemase OXA-51.

### Staffing

Patients collective on the NIMCU includes neonates needing special neonatal care as defined in recent literature [28] and neonates needing standard paediatric care. On the herein described NIMCU, nurse:patient ratio was intended to follow present recommendations. For 18 patients, the shift teams were composed of at least 3 board certificated neonatal experienced nurses and mostly 1 trainee nurse. During OV, nurse:patient ratio was increased as described below.

### Patients’ characteristics on the neonatal intermediate care unit

Patient’s entry criteria for NIMCU were a stable circulatory constitution and respiratory status without the need of invasive ventilation neither non-invasive continuous positive airway pressure (CPAP).

At NIMCU, continuous monitoring of heart- and breathing rate and oxygen saturation was performed. Supplementary (high flow) oxygen was available. Alimentation was performed with total or partial parenteral, nutrition via nasogastric tube, baby bottle or breast feeding. Parenteral nutrition was given via central or peripheral venous lines. Feeding of mothermilk and breastfeeding was preferred and supported. During OV and POST-OV, no patient had either external liquor drainage or arterial inserted cannulas.

### Breast feeding policy on the neonatal intermediate care unit

Nutrition with mother milk and breast feeding is a definitive goal at NIMCU. Staff team members and mothers were instructed and supported by an international board-certificated lactation nurse. In case of MDRGN positive mother, the mother was instructed to use a separate milk pump; her milk was stored in a separate fridge with the intention to avoid MDRGN transmission of potential MDRGN containing mother milk via external contamination of the mothermilk containing baby bottles. If the baby’s condition allowed breastfeeding, it was performed like in MDRGN negative mothers. Scheduled MDRGN screening of mothermilk was not done.

### Neonatal intermediate care unit staff’s hygiene training

All neonatal staff team members are being trained in hygiene management of neonates at least twice a year. Additionally, one senior pediatrician at NIMCU is designated as duty hygiene officer (DHO) to supervise hygiene procedures. In any case of suspected increase of MDRGN incidence on the ward, an additional ward round was immediately held with staff of the Institute for Medical Microbiology and Infection Control (IMMIC) of UHF in order to evaluate hygiene procedures on the ward,. Further advice or assistance was constantly given by staff of the IMMIC. During OV and POST-OV, no additional staff team training was provided.

### Management of multidrug-resistant gram-negative organism-colonized patients

Care of MDRGN colonized patient was provided by staff wearing gloves and gowns according to the current recommendations by the Commission for Hospital Hygiene and Infection Prevention of the Robert Koch Institute, Berlin, Germany (KRINKO) [29]. In case of detection of *Enterobacteriaceae* with extended spectrum beta–lactamase (ESBL)–phenotype, and *Enterobacteriaceae, Acinetobacter baumannii* and *Pseudomonas aeruginosa* resistant to piperacillin and any 3rd/4th generation cephalosporin or 2MRGN, the patient was not separated in single room. Parents need not wear gloves and gowns but were trained in hands hygiene, repeatedly. In case of detection of an aforementioned species with additional resistance to fluoroquinolones and / or to carbapenems, patient was separated in single room or bed-to-bed distance was arranged to 3 m at least. In these cases, parents were strictly advised to wear gloves and gowns.

### Visitors policy and training

Parents were encouraged to visit their neonate 24 h a day. The total amount of visitors/baby was limited to two persons. At their first visit, all visitors received a short instruction of the hygienic rules performed by the attending nurse.

### Microbiological surveillance for multidrug-resistant gram-negative organisms on the neonatal intermediate care unit

As announced by the KRINKO in 2012, microbiological screening for detection of multidrug-resistant organisms is recommended for patients on day of admission to NICU or NIMCU as well as weekly routine screening for patients treated at NICU or NIMCU in Germany [25, 26]. In order to intervene in case of any epidemiological shift exceeding the average MDRGN incidence on the ward, we furthermore set these findings in relation to the MDRGN incidence monthly calculated for the NIMCU since January 2014.

Patients admitted to the Department of Neonatology at UHF were screened for MDRGN, methicillin-resistant *Staphylococcus aureus* (MRSA) as well as vancomycin-resistant *Enterococcus faecalis/faecium* (VRE) by rectal and throat swabs. Weekly regular infectious disease interdisciplinary rounds were co-hosted by staff of the IMMIC as well as of department of Neonatology in order to ensure continuous active surveillance on the NIMCU.

### Molecular characterization of transmitted bacteria

For whole genome sequencing (WGS), DNA was isolated using the Purelink Genome DNA Mini kit (Thermo Fisher Scientific, Darmstadt, Germany) from overnight cultures according to the manufacturer’s instruction. WGS was carried out using an Illumina Nextera XT library with 2x150bp paired-end reads on an Illumina MiSeq instrument (Illumina, San Diego, CA, USA). The raw data was assembled using SPAdes (version 3.0) (1). The assembled contigs were analysed for multilocus sequence types using MLST 1.8 (2) and acquired antibiotic resistance genes using ResFinder (3).

### Statistical analysis

Average incidence of nosocomial MDRGN transmissions in the NIMCU was calculated based on the monthly nosocomial MDRGN incidence of the last 12 months of ward-regular bed occupancy (PRAE-OV). Chi squared test was performed for statistical analysis. 95% confidence intervals (95% CI) for frequencies were calculated based on binomial distribution and used to confirm statistical significance. *P*-value calculations were not used to evaluate statistical significance as it has been criticized for low reliability [30].

### Research process

This study was a prospective observational study escorting a high risk period on a neonatal intermediate care unit. Data were obtained and compared to pre- and post high risk period surveillance data. Major endpoint was incidence of MDRGN; variables included nurse to patient ratio and bed- to bed- distance.

## Results

### PRAE-overcrowding period

Based on the 12 months period prior to OV, mean number of patients admitted to the NIMCU was 35 per month, defined as the basic occupancy. During PRAE-OV, the average number of nosocomial transmission of MDRGN amounted to 2 per month, resulting in a cumulative incidence of 4.8% (95%CI = 2.9–7.3). Bed capacity was 18, nurse:patient ratio 1: 4.5–6 (*numeri integri* 3–4:18). Under these conditions, bed-to-bed distance amounted to 2 m. Parent-child contact was supported with more than 2 h of kangarooing/day.

### Overcrowding period

Number of NIMCU beds increased from 18 to 22. This resulted in a decrease of bed-to-bed distance to 1.0–1.2 m; kangarooing-time could not be continuously provided. Considering that the distance between bed-to-bed-distance is as been recommended to be 2 m at least [19–22], OV was suggested to be a risk setting for transmission of MDRGN. In contrast to PRAE-OV, these patients were merged in one room. Nurse:patient ratio was increased to 1: 4.4–5.5 (4–5:22) in terms of an increased number of board certificated neonatal nurses taking care for all patients with special neonatal care. One additional neonatal trainee nurse was implemented into patients care for babies without the need for special neonatal care. In the following, only the numbers of experienced nurses are mentioned. Numbers of ward physicians or consultants remained unchanged as well as teaching schedules for nurses or students.

In total, 43 patients with a mean birthweight of 2120 g (mean; range 490–4340 g) were treated on the NIMCU during OV. The ward occupancy therefore increased by almost 147% compared to the basic occupancy during PRAE-OV. Treatment of 20/43 patients extended into POST-OV.

7 patients were tested positive for MDRGN (Table 1). In two patients (B and E; Table 1) potential nosocomial transmission of MDRGN was observed. Nosocomial transmission of patient E is a particular case. Mother of baby C was tested positive for *Enterobacter cloacae* with resistance to 3rd/4th generation cephalosporins (*E. cloacae* Ceph) prior to birth of her baby. She vertically transmitted the strain to her own child and additionally was involved in nosocomial transmission of *E. cloacae* Ceph to baby E. This nosocomial transmission of *E. cloacae* Ceph to child E was most likely associated to misbehavior of mother C as she provoked direct close skin-to-skin contact to baby E for several times. One week after skin contact between mother C and baby E, baby E was tested positive for *E. cloacae* Ceph for the first time. Resistance pattern of the *E. cloacae* Ceph detected in baby E were identical to the *E. cloacae* Ceph strains detected in mother C and baby C which might indicate bacterial transmission from mother C to baby E.

**Table 1:** Patients tested positive on NIMCU during OV

Patient^a^	Sex	MDRGN species	Mode of acquisition
A	m	*Escherichia coli* ESBL	vertical
B	m	*Enterobacter aerogenes* Ceph	nosocomial
C	m	*Enterobacter cloacae* Ceph	vertical
D	f	*Enterobacter cloacae* Ceph	vertical
E	m	*Enterobacter cloacae* Ceph	nosocomial transmitted to baby E by mother of baby C
F	f	*Enterobacter cloacae* Ceph	vertical

^a^numbered consecutively. *Abbreviations: m* male, *f* female, *Ceph* resistance to 3rd/4th generation cephalosporin, *ESBL* extended spectrum beta-lactamase

In total, cumulative incidence of nosocomial MDRGN transmissions amounted to *n* = 2 in 43 patients, resulting in 4.7% (0.6–15.8) in OV. This did not significantly differ from the cumulative incidence of nosocomial MDRGN transmissions during PRAE-OV (4.8%; 2.9–7.3).

### POST-overcrowding period

In total, 41 patients were treated on NIMCU during POST-OV. Ward occupancy was almost 125%, slightly exceeding the basic occupancy during PRAE-OV. 7 patients were tested positive for MDRGN (Table 2), with two of them being residuals from OV (A and F), one pair of twins (H_twin1_ and H_twin2_) and two vertical transmissions (I and J) - similarly.

**Table 2:** Patients tested positive on NIMCU during POST-OV

Patient^a^	Sex	MDRGN species	Mode of acquisition
A	m	*Escherichia coli* ESBL	vertical
F	f	*Enterobacter cloacae* Ceph	vertical (OV)
G	m	*Enterobacter cloacae* Ceph	nosocomial
H_twin1_	m	*Citrobacter freundii* Ceph	vertical
H_twin2_	m	*Citrobacter freundii* Ceph *Acinetobacter baumannii*	vertical
I	m	*Escherichia coli* ESBL	vertical
J	m	*Escherichia coli* ESBL/FQ	vertical

^a^numbered consecutively. *Abbreviations: m* male, *f* female, *Ceph* resistance to 3rd/4th generation cephalosporin, *ESBL* extended spectrum beta-lactamase, *ESBL/FQ* ESBL + resistance to fluoroquinolones

Nosocomial transmission of *E. cloacae* Ceph (patient G; Table 2) occurred in one case. Number of nosocomial MDRGN transmissions amounted to *n* = 1 in 41 patients, resulting in a cumulative incidence of 2.4% (0.0–12.9). This did not significantly differ from the cumulative incidence of nosocomial MDRGN transmissions during PRAE-OV (4.8%; 2.9–7.3) and OV (4.7%; 0.6–15.8).

### Whole genome sequencing results

The *Enterobacter cloacae* isolates from the transmission event were whole genome sequenced to investigate their identity. They depicted an identical new multilocus sequence type (allele variants depicted in Table 3), and carried both the AmpC beta-lactamase *bla*_ACT-7_ and the fosfomycin resistance gene fosA, indicating a clonal spread.

**Table 3:** Multilocus variants found in *Enterobacter cloacae* Baby C and Baby E

Allele	Baby C	Baby E
*dnaA*	49	49
*fusA*	69	69
*gyrB*	20	20
*leuS*	65	65
*pyrG*	64	64
*rplB*	4	4
*dnaA*	6	6

## Discussion

In this study, we implemented the available recommendations concerning neonatal MDRGN transmission into the daily life settings on NIMCU in the scenario of a short-term and high risk overcrowding period.

### Infection control

Overcrowding has formerly been described as a promotor of nosocomial transmission for several pathogens [12–17, 29]. Considering that the NIMCU at UHF was facing a 25-day-overcrowding-period, we prepared in several ways for this precarious period. Prior to OV the hygiene risk associated with this situation was diligently discussed with the NIMCU staff. Parents received fundamental information as well. NIMCU staff underwent scheduled infection control training on e.g. correct hands hygiene during PRAE-OV, OV and POST-OV. This training was held repeatedly and multidisciplinary by the DHO and by staff of the IMMIC of UHF. Thus, these training units did not exceed the scheduled ones.

Low-threshold and broad access to all issues regarding aspects of infection control arising from OV and POST-OV was provided. Feedback was given immediately, precisely and constructively in any case, either in a positive or negative respect.

Incidence of MDRGN on our NIMCU did not increase during the OV. We therefore assume our management consisting of active surveillance, staff training as well as adherence to infection control demands has proven to be a successful procedure to escort through overcrowding period and prevent the spread of multidrug-resistant organisms in hospital setting. Nevertheless, according to other influencing factors like staff turnover and to maintain continuous alertness; training, bed side supervision and active surveillance have to be implemented as a substantial part in the care of this challenging patient group not only in special risk situations like overcrowding.

### Staffing

Recently published recommendations for perinatal centers [28] suggest a nurse:patient ratio of 1:1 for neonatal intensive treatment patients, 1:2 for neonatal intensive monitored patients (e.g. those with non-invasive ventilation, CVC, pleural drainage) and 1:4 for neonatal special care. In NICUs, caring of more than two patients by the same nurse increases the risk of nosocomial infections (NI) clearly [16, 21].

Ventilatory or circulatory support as well as necessity of CVCs or other invasive catheters is less likely in NIMCU than in NICU patients implicating fewer patients to staff contact and less risk for NI.

However, patients’ immunological maturation is still ongoing and host defence against bacteria remains fragile [31]. Prematurity related apnoea-bradycardia-syndrome with the need of frequent tactile stimuli is one of the predominant diseases of the NIMCU patients and regular care procedures are performed 6 to 12 times per day. Moreover, ultrasound examinations are regularly provided and additional patient to staff contact is common e.g. because of teaching issues. The number of patient to staff contacts of an NIMCU patient might therefore exceed the number during his NICU period.

Given this characteristics of a NIMCU patient, the takeover of NICU recommendations for NIMCU seems reasonable, but does not represent daily routine on NIMCU. In our setting, we aimed to reach the nurse:patient ratio of 1:4 at least in the high risk overcrowding period. Though we did not reach the recommended ratio regarding the total amount of NIMCU patients, we were able to provide the 1:4 ratio in terms of one experienced board certificated neonatal nurse for four neonates receiving special care. As this staff management together with the microbiological surveillance mentioned above resulted in an unaltered MDRGN-transmission rate during OV, we were able to underline the pivotal role of adequate staffing on NIMCUs or NICUs.

### Bed to bed distance

An appropriate bed to bed distance seems reasonable to allow easy staff movements around the incubator without contaminating the opposite patient with non-airborne MRE. The question, how narrow would be enough to prevent transmission is not examined, however, the literature recommend 2 m bed to bed distance for NICUs [32–34]. Moreover, if kangarooing should be provided, 2 m space is needed to place deckchairs and to allow parents enough space. During OV, kangarooing was less likely and parental presence between two beds was limited. In this special setting, bed-to-bed distance of 1–1.2 m seemed to be sufficient in terms of preventing MRE transmission.

### Impact of visitors / parents

Our results also point out the impact of parental carriage of MDRGN. Considering that 8 to 16% of the adult Rhine-Main population is colonized with MDRGN [7, 35], we suggest that parents might serve a risk in terms of infection control.

Although pointed out by several information boards and easy accessible dispensers for hand disinfection, hospital visitors seem to be still less aware on the impact of hand hygienic discipline [36]. Limiting access for visitors but not for parents therefore seems reasonable. Regular visits of parents with intense skin to skin contact (e.g. kangooroing) to their newborn child are inevitable for adequate bacterial skin and gut colonization [31].

Though, paternal MDRGN colonization will not be recognized as fathers are not screened and therefore serve as microbiological ‘black box’. Nevertheless, even if maternal MDRGN colonization is emerged, many questions may arise in terms of adequate preventive policy, e.g. the management of mother milk remain. Data on incidence, time course, and outcome of MDRGN transmission by breast milk are not available and prospective studies will not be performed due to ethical reasons. Thus, individual adjusted risk management, weighing the benefits of mother milk nutrition for the individual patient (e.g. reduced risk for necrotizing enterocolitis (NEC)) against the probability of MDRGN transmission (either vertical or nosocomial) and potential biological hazard, especially in case of colonization with MDRGN, is recommended [25, 26, 29].

The consideration whether mother milk and breast feeding could be the key incident in materno-fetal (and potential nosocomial) MDRGN transmission cannot be answered up to now and remains critical, particularly in high risk situations such as overcrowding. Impressingly, our study demonstrated that language skills, intellectual or cultural causes or simple lack of time might lead to a violence of hygienic rules; however,, the final result might be nosocomial MDRGN transmission. The transmission of *E. cloacae* Ceph between baby C and baby E has been verified via molecularbiological techniques. This transmission event shows that unexpected parental misbehavior – e.g. due to language barriers – can counteract any attempt of infection control. This, however, is an immutable factor but shows all the more that infection control is a team game being never at rest [37–39].

Our results might be biased by the fact, that child-to-parent contact including kangooroing was reduced due to less space between beds. Close skin-to-skin contact might increase the MDRGN transmission from parents to their newborn baby. Given the baseline incidence of MDRGN carrier in Germany, there is a theoretical risk for paternal-child bacterial transmission that was probably reduced by limitation of kangarooing during our OV setting.

## Conclusion

The key issues in prevention of nosocomial spread of MDRGN on NIMCUs during overcrowded periods of a NIMCU seems to be an adequate nurse:patient ratio, staff training and concise microbiological surveillance which should be guaranteed to escort through overcrowding periods. In our setting, bed-to-bed distance might not have played a key role. We think that the role of parental MRDGN transmission, transmission via breast milk in particular, and their impact on infection control management on neonatal departments should be in the focus of future investigations.

## Abbreviations

CEO: Chief Executive Officer
CI: Confidence interval
CLSI: Clinical and Laboratory Standards Institut
CPAP: Continuous Positive Airway Pressure
CVC: Central Venous Lines
CW: Calendar week
DHO: Duty Hygiene Officer
ESBL: Extended-Spectrum Beta–Lactamase
IMC: Intermediate Care Unit
IMMIC: Institute for Medical Microbiology and Infection Control
KRINKO: Commission for Hospital Hygiene and Infection Prevention of the Robert Koch Institute, Berlin, Germany
MALDI–TOF: Matrix-assisted–laser desorption ionization–time of flight analysis
MDRGN: Multidrug resistant Gram-negative bacteria
MERS-CoV: Middle East Respiratory Syndrome coronavirus
MRSA: Methicillin resistant *Staphylococcus aureus*
NI: Nosocomial infection
NICU: Neonatal Intensive Care Unit
NIMCU: Neonatal Intermediate Care Unit
NU: Neonatal Units
OV: Overcrowding period
POST-OV: Post Overcrowding period
PRAE-OV: Period prior to OV
SARS: Severe Acute Respiratory Syndrome
SARS-CoV: Severe Acute Respiratory Syndrome Coronavirus
UHF: University Hospital Frankfurt
VRE: Vancomycin resistant *Enterococcus* spp.
